# Efficacy and safety of the muscarinic receptor agonist KarXT (Xanomeline-Trospium) in schizophrenia: a systematic review, meta-analysis and Bayesian sensitivity analysis

**DOI:** 10.1093/ijnp/pyaf045

**Published:** 2025-07-07

**Authors:** Nikhil Sharma, Mahalaqua N Khatib, Roopashree R, Mandeep Kaur, Manish Srivastava, Amit Barwal, Garbham Venkata Siva Prasad, Pranchal Rajput, Vinamra Mittal, Muhammed Shabil, Amit Kumar, Ganesh Bushi, Rachana Mehta, Prakasini Satapathy, Sanjit Sah

**Affiliations:** Department of Pharmacy Practice, National Institute of Pharmaceutical Education and Research, Guwahati 781101, India; Division of Evidence Synthesis, Global Consortium of Public Health and Research, Datta Meghe Institute of Higher Education, Wardha, India; Department of Chemistry and Biochemistry, School of Sciences, JAIN (Deemed to be University), Bangalore, Karnataka, India; Department of Allied Healthcare and Sciences, Vivekananda Global University, Jaipur, Rajasthan 303012, India; Department of Endocrinology, NIMS University, Jaipur, India; Department of Pharmacy, Chandigarh Pharmacy College, Chandigarh Group of College, Jhanjeri, Mohali, Punjab 140307, India; Department of Chemistry, Raghu Engineering College, Visakhapatnam, Andhra Pradesh 531162, India; School of Applied and Life Sciences, Division of Research and Innovation, Uttaranchal University, Dehradun, India; Graphic Era Institute of Medical Sciences, Graphic Era (Deemed to be University), Clement Town, Dehradun, India; University Center for Research and Development, Chandigarh University, Mohali, Punjab, India; Medical Laboratories Techniques Department, AL-Mustaqbal University, Hillah, Babil 51001, Iraq; Centre of Research Impact and Outcome, Chitkara University, Rajpura, Punjab 140417, India; Chitkara Centre for Research and Development, Chitkara University, Himachal Pradesh 174103 India; School of Pharmaceutical Sciences, Lovely Professional University, Phagwara, India; Clinical Microbiology, RDC, Manav Rachna International Institute of Research and Studies, Faridabad, Haryana 121004, India; Center for Global Health Research, Saveetha Medical College and Hospital, Saveetha Institute of Medical and Technical Sciences, Saveetha University, Chennai, India; University of Cyberjaya, Persiaran Bestari, Cyber 11, Cyberjaya, Selangor Darul Ehsan 63000, Malaysia; SR Sanjeevani Hospital, Kalyanpur, Siraha 56517, Nepal; Department of Paediatrics, Dr. D. Y. Patil Medical College Hospital and Research Centre, Dr. D. Y. Patil Vidyapeeth (Deemed-to-be-University), Pimpri, Pune, Maharashtra 411018, India; Department of Public Health Dentistry, Dr. D.Y. Patil Dental College and Hospital, Dr. D.Y. Patil Vidyapeeth (Deemed-to-be-University), Pune, Maharashtra 411018, India

**Keywords:** KarXT, meta-analysis, randomized controlled trials, schizophrenia, systematic review, trospium xanomeline

## Abstract

**Background:**

Schizophrenia significantly impacts global health, with existing treatments primarily focusing on positive symptoms and often causing considerable side effects. KarXT (Xanomeline-Trospium), a novel treatment combining xanomeline, a muscarinic M1/M4 receptor agonist, and trospium, targets a broader range of symptoms including negative and cognitive deficits, potentially with fewer side effects. This systematic review and meta-analysis evaluates the efficacy and safety of KarXT in treating schizophrenia, assessing symptom reduction and safety profiles compared to placebo.

**Methods:**

We searched PubMed, Embase, and Web of Science up to November 10, 2024, for randomized controlled trials (RCTs) assessing the efficacy and safety of KarXT in schizophrenia. Data were pooled using a random-effects model, assessing outcomes like Positive and Negative Syndrome Scale (PANSS) scores and incidence of treatment-emergent adverse events (TEAEs). R software (Version 4.4.) was used for meta-analysis.

**Results:**

Three RCTs involving 674 participants were included. KarXT significantly reduced total PANSS scores of mean difference (MD) –9.707 (95% CI: –12.329 to –7.085), with notable improvements in both negative (MD = –1.623; 95% CI: –2.461 to –0.785) and positive symptom subscales (MD = –3.213; 95% CI: –4.033 to –2.393). The treatment was associated with a higher incidence of TEAEs, predominantly constipation [risk ratio (RR): 2.77; 95% CI: 1.72–4.45] and nausea (RR: 4.87; 95% CI: 2.73–8.68) compared to placebo. Bayesian meta-analysis confirmed the results.

**Conclusion:**

KarXT offers significant improvements in both negative and positive symptoms of schizophrenia with a manageable safety profile. Its potential as a transformative treatment for schizophrenia highlights the need for further research to confirm these findings and to fully understand its long-term efficacy and safety.

## INTRODUCTION

Schizophrenia is a chronic, severe psychiatric disorder that affects ⁓23.6 million of the global population.^1^ Characterized by a constellation of symptoms, including positive (hallucinations, delusions), negative (blunted affect, social withdrawal), and cognitive deficits (impaired memory, attention, executive function),^2^ schizophrenia presents significant challenges to individuals and healthcare systems alike. The condition is often debilitating and can result in a decreased quality of life, diminished functioning, and increased morbidity and mortality rates. The management of schizophrenia primarily involves pharmacological intervention, with antipsychotic medications being the cornerstone of treatment.^3^ However, current antipsychotic drugs, though effective in controlling positive symptoms, are often accompanied by significant side effects, particularly extrapyramidal symptoms (EPS), weight gain, and metabolic disturbances.^4^ Additionally, antipsychotic drugs do not address negative symptoms and cognitive impairments adequately, which remain a major source of disability for many patients with schizophrenia.

In recent years, there has been growing interest in exploring novel pharmacological agents that target different aspects of the brain’s neurotransmitter systems, including those that regulate cholinergic activity. The muscarinic acetylcholine receptors (mAChRs) are a group of G-protein-coupled receptors that play critical roles in various cognitive and emotional processes, and disturbances in cholinergic signaling have been implicated in the pathophysiology of schizophrenia The muscarinic acetylcholine receptors (mAChRs) are a group of G-protein-coupled receptors that play critical roles in various cognitive and emotional processes, and disturbances in cholinergic signaling have been implicated in the pathophysiology of schizophrenia.^5^ Muscarinic receptor dysfunction has been proposed to contribute to cognitive and negative symptoms, and thus, targeting this system may offer new avenues for therapeutic intervention.^6^

One promising pharmacological approach that has garnered attention is the combination of xanomeline, a selective muscarinic M1/M4 receptor agonist, and trospium, a peripheral muscarinic antagonist.^7^ This combination, known as KarXT (xanomeline–trospium), is designed to maximize central muscarinic receptor activation while minimizing peripheral side effects such as gastrointestinal disturbances and bradycardia.^8^ The potential of KarXT to improve cognitive and negative symptoms in schizophrenia while avoiding the common side effects associated with traditional antipsychotics has been investigated in several randomized controlled trials (RCTs). This combination therapy aims to restore the balance of cholinergic signaling in the brain, specifically targeting the M1 receptor, which is thought to play a crucial role in cognitive function, and the M4 receptor, which is associated with the regulation of dopamine release and the modulation of the negative symptoms of schizophrenia.^9^

While the dual-agonist approach may offer advantages in terms of efficacy, there are concerns about its safety profile, particularly given the challenges associated with manipulating the cholinergic system. Adverse effects such as muscarinic toxicity, including dry mouth, blurred vision, and urinary retention, may still occur, albeit at a lower incidence due to the presence of the peripheral muscarinic antagonist trospium.^10^ The need for new and effective treatments for schizophrenia is urgent, as current pharmacotherapies often fail to address the full spectrum of symptoms experienced by patients. Cognitive and negative symptoms remain particularly resistant to treatment, and their persistence contributes significantly to the overall burden of the disease. Given these uncertainties, a comprehensive synthesis of the available evidence is needed to better understand the potential benefits and risks of this novel pharmacological treatment.

This systematic review and meta-analysis aims to evaluate the efficacy and safety of KarXT in the treatment of schizophrenia. By analyzing data from RCTs, we seek to assess the therapeutic effects of KarXT on key outcomes such as symptom reduction (both positive and negative), including the safety profile of KarXT by examining the incidence of common adverse events, particularly those related to the muscarinic system. In doing so, we hope to provide a clearer picture of the potential role of KarXT in the clinical management of schizophrenia, comparing it to other treatment options currently available, and informing future research directions.

## METHODS

### Study Design

This systematic review and meta-analysis were conducted to evaluate the efficacy and safety of the muscarinic receptor agonist KarXT in the treatment of schizophrenia. The study adhered to the guidelines outlined in the Preferred Reporting Items for Systematic Reviews and Meta-Analyses (PRISMA) statement (Table S1). A pre-registered protocol detailed the study objectives, eligibility criteria, and statistical approach to ensure transparency and reproducibility.

### Eligibility Criteria

We included RCTs that investigated the efficacy and safety of KarXT in individuals (≥18 years) diagnosed with schizophrenia based on standardized diagnostic criteria (e.g. DSM-5, ICD-10). Eligible studies were required to report quantitative outcomes related to efficacy, e.g. symptom severity using standardized scales such as Positive and Negative Syndrome Scale (PANSS) and safety, e.g. treatment-emergent adverse event (TEAE). Studies were included regardless of publication status or language, provided sufficient data were available for analysis. Observational studies, case reports, and non-randomized trials were excluded.

### Search Strategy

A comprehensive literature search was conducted in the following electronic databases: PubMed, Embase, web of science (WOS). The search strategy used Medical Subject Headings free-text terms combining “KarXT,” “xanomeline,” “trospium,” “schizophrenia”. The search was performed from database inception to 10 November 2024. Boolean operators (AND, OR) were applied to refine the search. The complete search strategy is present in Table S2. No restrictions were placed on publication date. Additionally, manual search was also conducted.

### Study Selection and Screening

To aid in the study selection process, we utilized semi-automated software (Nested-Knowledge, MN, USA) to de-duplication and screen the studies. After removing duplicates, titles and abstracts were screened independently by two reviewers (blind to each other’s assessments) to identify studies meeting the eligibility criteria. Full texts of potentially relevant articles were retrieved and evaluated for inclusion. Discrepancies were resolved through discussion or consultation with a third reviewer.

### Data Extraction

A standardized data extraction form was used to collect study characteristics, including author information, publication year, sample size for both intervention and control groups, patient demographics, diagnostic criteria, intervention details (dose of KarXT), control conditions (placebo or active comparator), outcome measures, and adverse events. Population characteristics such as mean age, baseline PANSS and Clinical Global Impressions (CGI) score for both intervention and control groups were also gathered. Data were extracted independently by two reviewers, and disagreements were resolved through consensus or a third-party adjudication.

### Risk of Bias Assessment

The methodological quality of included studies was assessed using the Cochrane Risk of Bias for randomized trials (RoB 2). Domains evaluated included randomization process, deviations from intended interventions, missing outcome data, measurement of the outcome, and selection of the reported result. Each study was rated as having a low, some concerns, or high risk of bias. Disagreements between reviewers were resolved by discussion or referral to a third reviewer.

### Data Synthesis and Statistical Analysis

A meta-analysis was conducted using random (I^2^ > 50%) and common effects (I^2^ < 50%) model to account for heterogeneity among studies. Pooled effect sizes were calculated mean differences (MD) with 95% confidence intervals (CIs) for efficacy outcomes including MD from baseline for PANSS total scores, positive subscale score, negative subscale score, marder negative factor score and CGI-S score, while dichotomous outcomes, such as adverse events, were presented as risk ratios (RR) with 95% CIs. Heterogeneity was assessed using the I^2^ statistic, with thresholds of 25%, 50%, and 75% indicating low, moderate, and high heterogeneity, respectively. Sensitivity analyses were performed to explore the robustness of findings.

We estimated the posterior probability of benefit reduction in PANSS scores with 95% Credibility intervals (Crl). All analyses used in this Bayesian framework were conducted using “vague priors,” which assume minimal prior knowledge about the parameters of interest, allowing the data to predominantly drive the posterior estimates while still incorporating a modest degree of regularization to stabilize the model. A Bayesian hierarchical random-effects model was applied. Primary Bayesian analyses were conducted using a vauue prior for the overall effect size μ ~ N(0, 100) and a half-Cauchy prior for heterogeneity τ ~ HC(0, 0.5). Between-study heterogeneity was evaluated using τ. Distributions for each study, derived from the hierarchical model, were graphically presented based on model estimates. Posterior probabilities were estimated using Markov Chain Monte Carlo (MCMC) sampling algorithms in R Statistics (Version 4.4; R Foundation for Statistical Computing, Vienna, Austria, https://www.R-project.org/), employing the “brms” package, with four chains and 10 000 saved iterations per chain. Posterior predictive checks were performed by simulating random draws from the posterior and comparing these replications to the observed data. Convergence of MCMC sampling was diagnosed based on trace plots and density plots. The results were presented as a joint posterior density plot of (effect parameter) and τ (heterogeneity parameter). All statistical analyses were performed using R software version 4.4.^11^

### Quality of Evidence

The Grading of Recommendations, Assessment, Development, and Evaluation (GRADE) approach was used to assess the quality of evidence for each outcome. Domains evaluated included risk of bias, inconsistency, indirectness, imprecision, and publication bias. The quality of evidence was categorized as high, moderate, low, or very low.^12^^,^^13^

## RESULTS

### Study Selection

**Figure 1:**
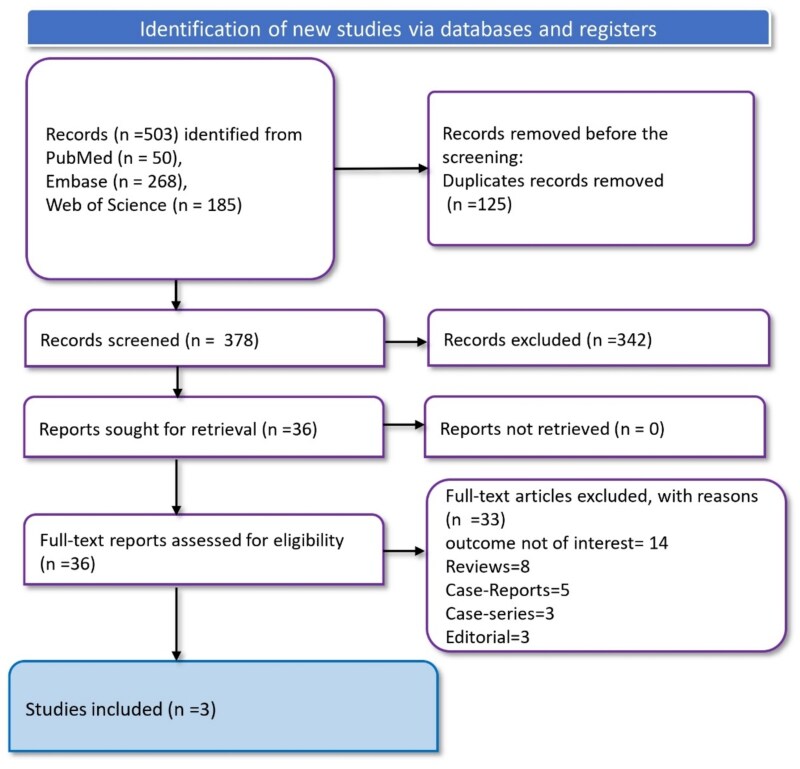
PRISMA flowchart depicting article selection and screening process. PRISMA, Preferred Reporting Items for Systematic Reviews and Meta-Analyses.

The systematic search identified three RCTs that met the inclusion criteria and were included in the analysis. A total of 503 records were initially retrieved from databases, including PubMed, Embase, and Web of Science. After removing 125 duplicates, 378 records were screened, of which 342 were excluded based on titles and abstracts. A total of 36 full-text articles were assessed for eligibility, with 33 excluded (Table S3) for reasons such as irrelevant outcomes (n = 14), reviews (n = 8), case reports (n = 5), case series (n = 3), and editorials (n = 3) (Figure 1).

### Study Characteristics

The included studies were randomized, double-blind trials conducted in the United States, focusing on the efficacy and safety of KarXT (xanomeline-trospium) for schizophrenia. Participants had a confirmed DSM-5 diagnosis of schizophrenia. Sample sizes ranged from 90 to 125 for KarXT groups and 92 to 131 for placebo groups, with mean ages between 43 and 46 years in both groups. All studies administered maximum dose of xanomeline 125 mg with trospium 30 mg. Baseline PANSS scores were comparable across groups, ranging from 97 to 98, and baseline CGI scores were similarly matched at ⁓5.0-5.1. These consistent designs and measures ensure the validity of the pooled analysis (Table 1). Summary of results has been presented in Table 2.

**Table 1:** Characteristics of included studies.

Study	Country	Study design	NCT	Inclusion criteria	Sample size (KarXT)	Sample size (Placebo)	Age (KarXT)	Age (Placebo)	Max Dose (KarXT)	Baseline PANSS score (KarXt)	Baseline PANSS score (Placebo)	Baseline CGI-score (KarXt)	Baseline CGI-score (Placebo)
**Brannan 2021** ^14^	USA	Randomized, double-blinded	NCT3697252	Patients had a primary diagnosis of schizophrenia	90	92	43.4 ± 10.1	41.6 ± 10.1	xanomeline 125 mg/trospium 30 mg	97.7 (SD = 9.7)	96.6 (SD = 8.3)	5.0 (SD = 0.6)	4.9 (SD = 0.6)
**Kaul 2023** ^15^	USA	Randomized, double-blinded	NCT04659161	Participants with a primary diagnosis of schizophrenia	117	119	45·9 ± 10·4	46·1 ± 10.8	xanomeline 125 mg/trospium 30 mg	98.2 (SD = 8.9)	97.7 (SD = 9.4)	5.1 (SD = 0.6)	5.1 (SD = 0.6)
**Kaul 2024** ^16^	USA	Randomized, double-blinded	NCT04738123	Adults aged 18-65 years with a diagnosis of schizophrenia	125	131	43.6 ± 11.4	42.6 ± 12.2	Xanomeline 125 mg/Trospium 30 mg	97.3 (8.9)	96.7 (SD = 8.9)	5.1 (SD = 0.6)	5.0 (SD = 0.6)

Abbreviations: CGI, Clinical Global Impressions; DSM5, Diagnostic and Statistical Manual of Mental Disorders, 5th Edition; KarXT, Xanomeline-Trospium; NCT, National Clinical Trial; PANSS, Positive and Negative Syndrome Scale; SD, Standard Deviation; USA, United States of America.

**Table 2:** Summary of Evidence of KarXT compared to Placebo for Schizophrenia.

Certainty assessment							Summary of findings				
Participants (studies) Follow-up	Risk of bias	Inconsistency	Indirectness	Imprecision	Publication bias	Overall certainty of evidence	Study event rates (%)		Relative effect (95% CI)	Anticipated absolute effects	
							With Placebo	With KarXT		Risk with Placebo	Risk difference with KarXT
**Pain and Negative syndrome scale scores (follow-up: mean 5 weeks)**											
**674 (3 RCTs)**	not serious	not serious	not serious	not serious	none	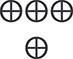 High	342	332	-	342	MD **9.707 Scores lower** (12.329 lower to 7.085 lower)
**PANSS negative subscale score (follow-up: mean 5 weeks; assessed with: Change in score)**											
**674** **(3 RCTs)**	not serious	not serious	not serious	not serious	none	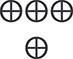 High	342	332	-	342	MD **1.623 Scores lower** (2.461 lower to 0.785 lower)
**PANSS positive subscale score (follow-up: mean 5 weeks)**											
**674** **(3 RCTs)**	not serious	not serious	not serious	not serious	none	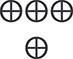 High	342	332	-	342	MD **3.213 Scores lower** (4.033 lower to 2.393 lower)
**PANSS marder negetive score (follow-up: mean 5 weeks)**											
**492** **(2 RCTs)**	not serious	serious^a^	not serious	serious^b^	none	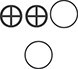 Low^a^^,^^b^	250	242	-	250	MD **1.466 score lower** (2.836 lower to 0.095 lower)
**Clinical Global Impression (follow-up: mean 5 weeks; assessed with: 7 point scale)**											
**492** **(2 RCTs)**	not serious	not serious	not serious	not serious	none	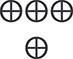 High	250	242	-	250	MD **0.584 score lower** (0.696 lower to 0.472 lower)
**Adverse events (follow-up: mean 5 weeks; assessed with: RR)**											
**683** **(3 RCTs)**	not serious	not serious	not serious	serious^c^	none	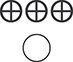 Moderate^c^	176/343 (51.3%)	231/340 (67.9%)	**RR 1.323** (1.144 to 1.530)	176/343 (51.3%)	**166 more per 1000** (from 74 more to 272 more)

CI, confidence interval; MD, mean difference; RR, risk ratio; PANSS, Positive and Negative Syndrome Scale; KarXT, Xanomeline-Trospium; RCTs, randomized controlled trials.

Explanations:

a High Heterogeneity detected.

b Confidence intervals overall with clinically non-significant level.

c Optimum information size not met due to less overall pooled sample size.

### Risk of Bias Assessment

**Figure 2:**
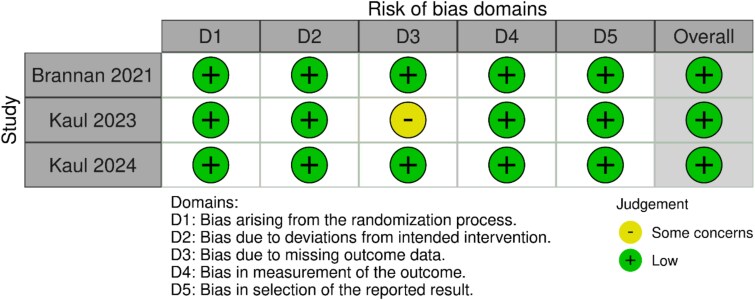
Risk of bias assessment.

The risk of bias assessment, performed using the Cochrane RoB 2 tool, indicated an overall low risk of bias across the included studies. One study (Kaul 2023)^15^ had “some concerns” regarding missing outcome data, but all other domains (randomization process, deviations from intended interventions, outcome measurement, and reporting) were rated as low risk (Figure 2).

### Efficacy Outcomes

#### PANSS Total Score

**Figure 3:**
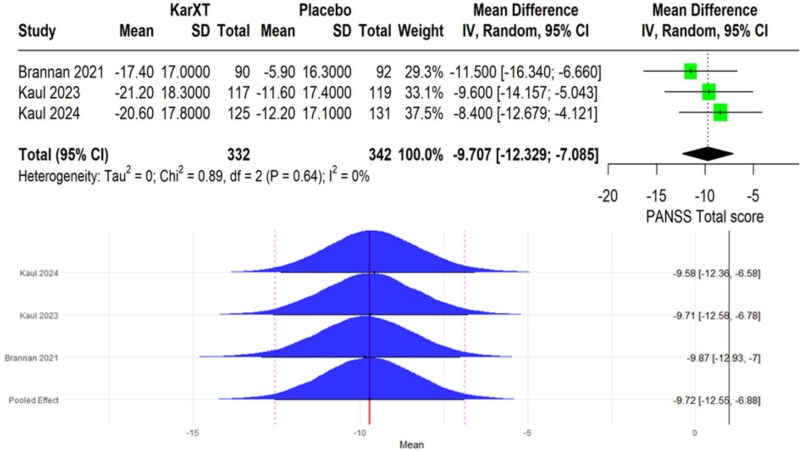
Forest plot showing the change in PANSS total score from baseline with KarXT. PANSS, Positive and Negative Syndrome Scale; KarXT, Xanomeline-Trospium.

The total PANSS score, which reflects the global severity of symptoms, was significantly reduced with KarXT compared to placebo (MD: –9.707; 95% CI: –12.329 to –7.085, *P* = .64), with no observed heterogeneity (I^2^ = 0%). These results underscore the broad therapeutic benefits of KarXT in addressing overall symptom burden in schizophrenia. Certainty of evidence for PANSS total score was found to be high (Figure 3). The Bayesian model showed a pooled MD of –9.72 with Crl ranging from –12.55 to –6.88. This ensured the robustness of result.

#### PANSS Negative Subscale

**Figure 4:**
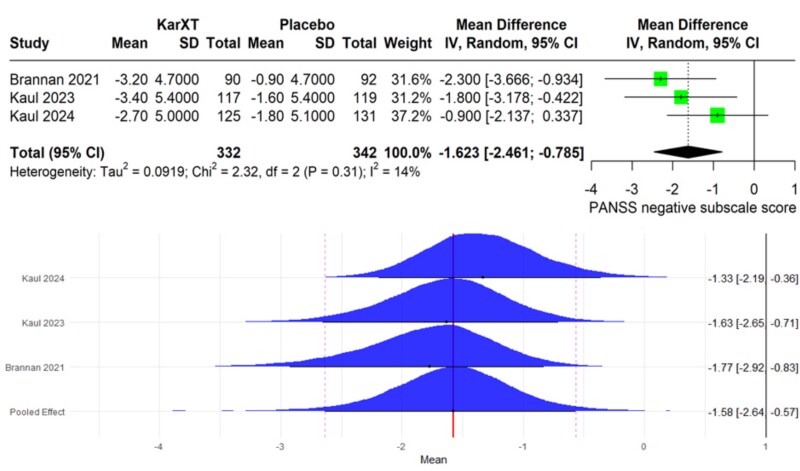
Forest plot showing the change in PANSS negative subscale score from baseline with KarXT. PANSS, Positive and Negative Syndrome Scale; KarXT, Xanomeline-Trospium.

The analysis of PANSS negative subscale scores demonstrated a significant mean difference of with KarXT compared to placebo (MD: –1.623; 95% CI: –2.461 to –0.785, *P* = .31). Heterogeneity was minimal (I^2^ = 14%), indicating consistent efficacy in alleviating negative symptoms across studies. This further confirms the specific effectiveness of KarXT in targeting negative symptomatology. Certainty of evidence for PANSS negative Subscale was found to be high (Figure 4). The Bayesian model showed a pooled MD of –1.58 (95% Crl: ranging from –2.64 to –0.57).

#### PANSS Positive Subscale

**Figure 5:**
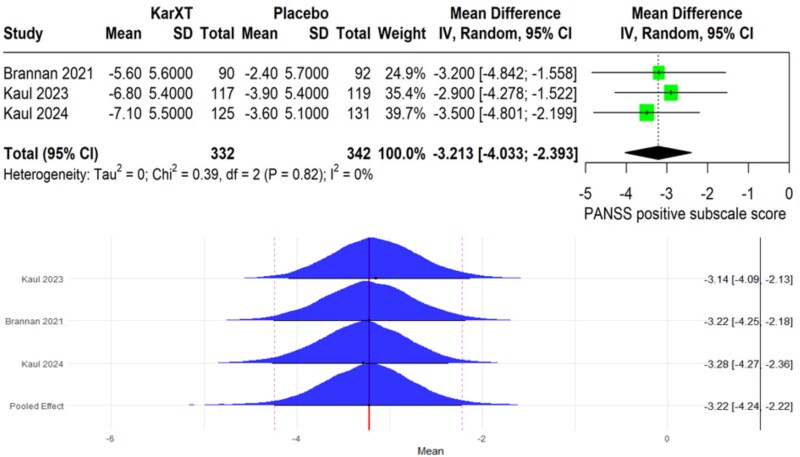
Forest plot showing the change in PANSS positive subscale score from baseline with KarXT. PANSS, Positive and Negative Syndrome Scale; KarXT, Xanomeline-Trospium.

The analysis of PANSS positive subscale scores showed a significant mean difference of favoring KarXT over placebo (MD: –3.213; 95% CI: –4.033 to –2.393, *P* = .82). Heterogeneity was low (I^2^ = 0%), suggesting consistent effects across studies. These findings support the effectiveness of KarXT in reducing positive symptoms in individuals with schizophrenia. Certainty of evidence for PANSS positive Subscale was found to be high (Figure 5). The Bayesian model showed a pooled MD of –3.22 (95% Crl: ranging from –4.24 to –2.22).

#### PANSS Marder Negative Subscale

**Figure 6:**
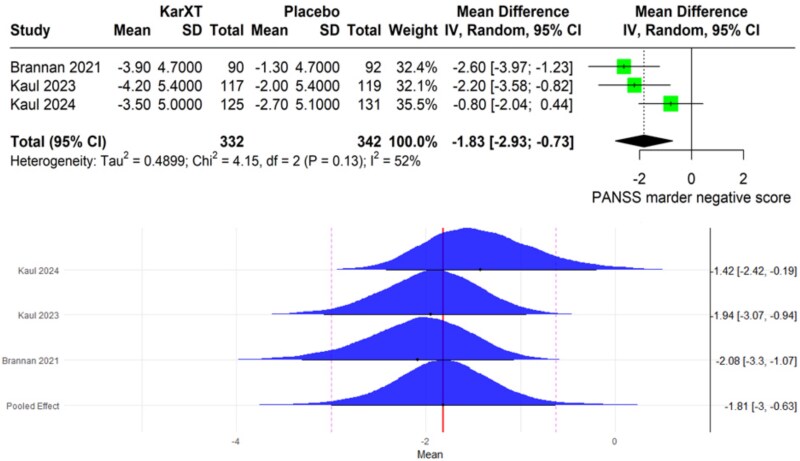
Forest plot showing the change in PANSS marder negative score from baseline with KarXT. PANSS, Positive and Negative Syndrome Scale; KarXT, Xanomeline-Trospium.

The meta-analysis demonstrated a significant improvement in PANSS Marder negative subscale scores with KarXT compared to placebo (MD: –1.83; 95% CI: –2.93 to –0.73, *P* = .13), favoring KarXT. Heterogeneity was moderate (I^2^ = 54%), indicating some variability in the effect size across studies. Certainty of evidence for PANSS Marder negative Subscale was found to be low (Figure 6). The Bayesian model showed a pooled MD of –1.81 (95% Crl: ranging from –3 to –0.63).

#### CGI Score

**Figure 7:**
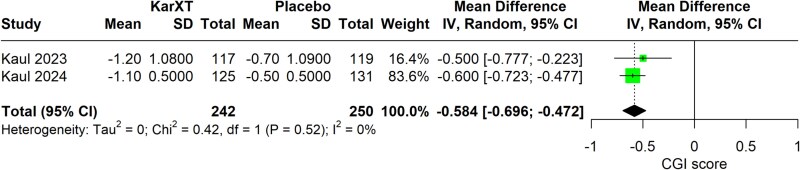
Forest plot showing the change in CGI score from baseline with KarXT. CGI, Clinical Global Impressions; KarXT, Xanomeline-Trospium.

The CGI score, reflecting the overall clinical improvement, showed a significant benefit of KarXT over placebo (MD: –0.584; 95% CI: –0.696 to –0.472, *P* = .52), with no heterogeneity (I^2^ = 0%). These findings provide robust evidence of KarXT’s clinical utility in improving overall patient outcomes in schizophrenia. Certainty of evidence for CGI score was found to be high (Figure 7).

#### Safety Outcomes

**Figure 8:**
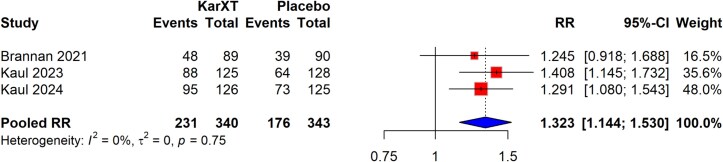
Forest plot showing the RR of TEAE associated with KarXT. RR, risk ratio; TEAE, treatment-emergent adverse event; KarXT, Xanomeline-Trospium.

**Figure 9:**
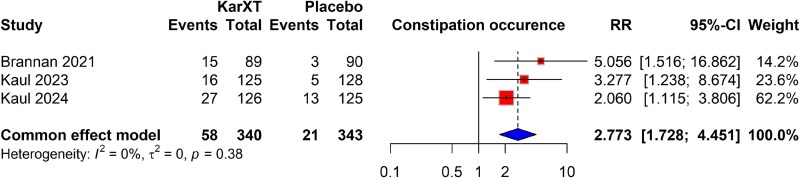
Forest plot showing the RR of constipation occurrence associated with KarXT. RR, risk ratio; KarXT, Xanomeline-Trospium.

**Figure 10:**
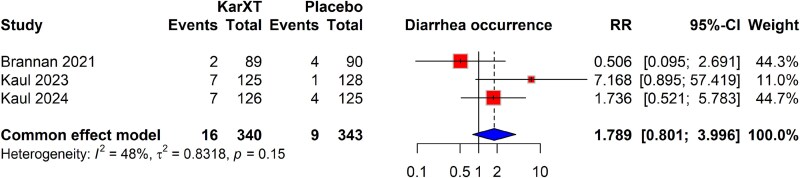
Forest plot showing the RR of diarrhea occurrence associated with KarXT. RR, risk ratio; KarXT, Xanomeline-Trospium.

**Figure 11:**
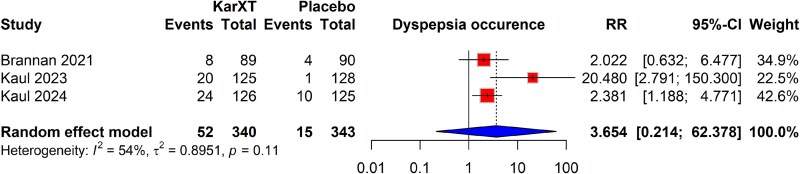
Forest plot showing the RR of dyspepsia occurrence associated with KarXT. RR, risk ratio; KarXT, Xanomeline-Trospium.

**Figure 12:**
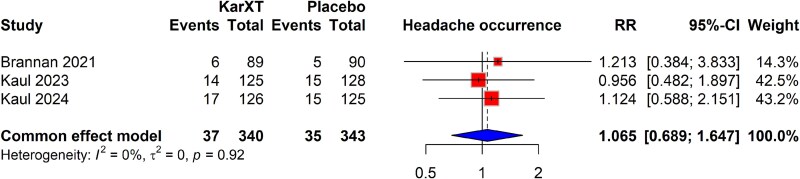
Forest plot showing the RR of headache occurrence associated with KarXT. RR, risk ratio; KarXT, Xanomeline-Trospium.

**Figure 13:**
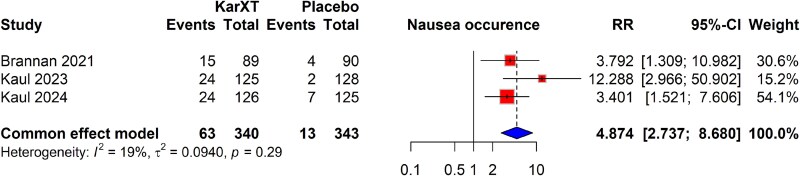
Forest plot showing the RR of nausea occurrence associated with KarXT. RR, risk ratio; KarXT, Xanomeline-Trospium.

**Figure 14:**
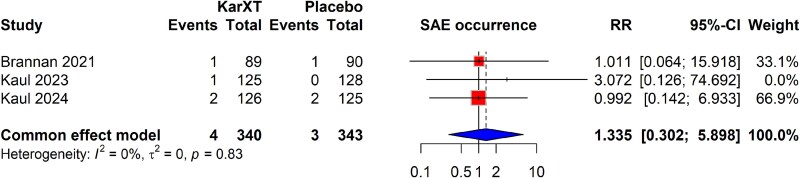
Forest plot showing the RR of SAE occurrence associated with KarXT. RR, risk ratio; SAE, serious adverse event; KarXT, Xanomeline-Trospium.

**Figure 15:**
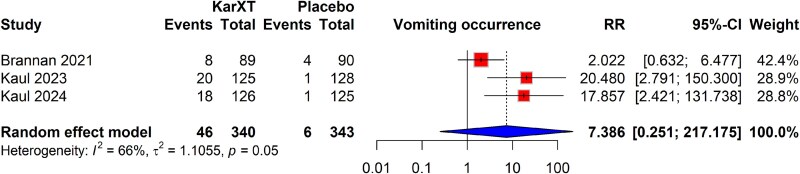
Forest plot showing the RR of vomiting occurrence associated with KarXT. RR, risk ratio; KarXT, Xanomeline-Trospium.

The safety analysis revealed a higher incidence of TEAEs in the KarXT group compared to placebo, (RR: 1.323; 95% CI: 1.144–1.530, *P* < .01), and no heterogeneity (I^2^ = 0%) (Figure 8). The safety profile of KarXT revealed an increased risk of constipation (RR: 2.773; 95% CI: 1.728-4.451), and no significant heterogeneity (I^2^ = 0%, *P* = .38) (Figure 9). The risk of diarrhea was lower with KarXT (RR: 1.789; 95% CI: 0.801-3.996), with moderate heterogeneity (I^2^ = 48%, *P* = .15) (Figure 10). Dyspepsia showed a notable increase with KarXT (RR: 3.654; 95% CI: 0.214-62.378), with moderate heterogeneity (I^2^ = 54%, *P* = .11) (Figure 11). The pooled RR for headache was 1.065 (95% CI: 0.689-1.647), indicating no significant difference between KarXT and placebo, with no heterogeneity (I^2^ = 0%, *P* = .92) (Figure 12). Nausea occurred more frequently with KarXT (RR: 4.874; 95% CI; 2.737-8.680), with low heterogeneity (I^2^ = 19%, *P* = .29) (Figure 13). The incidence of serious adverse events (SAEs) was comparable between the two groups (RR: 1.335; 95% CI: 0.302-5.898), with no heterogeneity (I^2^ = 0%, *P* = .83) (Figure 14). Finally, vomiting was more common in the KarXT group (RR: 7.386; 95% CI: 0.251-217.175), with moderate heterogeneity (I^2^ = 66%, *P* = .05) (Figure 15). These findings suggest that KarXT is associated with increased risks for constipation and nausea, while showing no significant impact on SAE, vomiting or headaches, with variable heterogeneity across the outcomes.

#### Sensitivity Analysis

A leave-one-out sensitivity analysis was conducted to assess the robustness of the results. The analysis confirmed that the pooled estimates for PANSS total score were stable, with the MD ranging from –8.962 to –10.493 across scenarios (Figure S1).

## DISCUSSION

The findings of this systematic review and meta-analysis provide robust evidence for the efficacy and safety of the muscarinic receptor agonist KarXT in the treatment of schizophrenia. By synthesizing data from three high-quality RCTs, we demonstrated that KarXT significantly improves both positive and negative symptoms of schizophrenia, as measured by standardized scales such as the PANSS and the CGI scale. The safety analysis further highlighted that, while treatment with KarXT is associated with an increased risk of TEAEs, these are largely tolerable and consistent with the pharmacological profile of the drug. Below, we contextualize these findings within the broader literature, explore potential mechanisms underlying the observed effects, and discuss implications for clinical practice and future research.

The pooled analyses revealed that KarXT significantly reduced PANSS total scores compared to placebo, with a MD of –9.707 (95% CI: –12.329 to –7.085; *P* < .01) and no observed heterogeneity (I^2^ = 0%). The observed MD exceeds the minimum clinically important difference (MCID) of 7–12 points for PANSS total scores established in prior studies.^17^^,^^18^ This suggests a clinically meaningful improvement in overall symptom severity. This substantial reduction indicates the broad therapeutic potential of KarXT in addressing schizophrenia symptoms. Importantly, KarXT also demonstrated statistically significant improvements in the PANSS negative subscale and PANSS positive subscale. The smaller MD for the PANSS negative subscale fall below the MCID thresholds for negative symptoms. While these improvements are statistically significant, their clinical relevance is less certain. These results are particularly notable given the historical difficulty in effectively treating negative symptoms, which are often resistant to conventional antipsychotics and contribute significantly to the functional impairment experienced by patients. While the observed reduction in PANSS Marder negative scores (MD = –1.466) was statistically significant, its clinical relevance remains uncertain, as the magnitude of change falls below the MCID using Clinical Antipsychotic Trials of Intervention Effectiveness (CATIE) trial data of 3–4 points established in prior studies.^17^ However, the moderate heterogeneity observed (I^2^ = 54%) suggests variability in treatment effects across studies, which warrants further investigation. Although the improvements in negative symptoms were modest and below conventional MCID thresholds, even small reductions in negative symptoms may hold clinical value given their resistance to current treatments. However, caution is warranted in interpreting these changes as transformative. CGI scores, which provide an overall assessment of symptom severity, also indicated consistent reductions with KarXT, reinforcing its broad-spectrum efficacy. Recent reviews also highlighted the efficacy of KarXT in improving schizophrenia symptoms, with significant reductions in PANSS total scores (MD –13.17; 95% CI –20.16 to −6.18; *P* = .0002) and improvements across all PANSS subscales.^19^ Additionally, Horan et al.^20^ demonstrated that KarXT significantly improves cognition in patients with baseline cognitive impairments (d = 0.54), independent of changes in psychotic symptoms.

The results of the Bayesian meta-analysis conducted to assess the efficacy of KarXT in treating schizophrenia demonstrated a striking similarity to the frequentist meta-analysis findings, reinforcing the robustness of the observed treatment effects. This concordance validates the efficacy of KarXT across multiple symptom domains, including positive and negative symptoms, and highlights the reliability of the Bayesian approach. The consistency between the two methodologies, despite the Bayesian model’s incorporation of priors, suggests that the data-driven evidence is sufficiently strong to overshadow prior influence, further supporting KarXT’s potential as a transformative treatment for schizophrenia.

KarXT’s dual mechanism of action likely underpins its therapeutic benefits. Xanomeline, the central muscarinic receptor agonist, selectively targets M1 and M4 receptors, which are implicated in key pathophysiological processes of schizophrenia.^21^ Activation of M1 receptors in the cortex and hippocampus is thought to enhance cognitive function and reduce negative symptoms by modulating acetylcholine signaling, while M4 receptor activation in the striatum regulates dopamine release, thereby attenuating positive symptoms.^22^ These pathways differentiate KarXT from conventional antipsychotics, which primarily act as dopamine D2 receptor antagonists and are associated with significant side effects and limited efficacy for negative and cognitive symptoms. The addition of trospium, a peripheral muscarinic antagonist, mitigates the systemic side effects commonly associated with muscarinic agonists, such as gastrointestinal disturbances and bradycardia.^23^ This peripheral antagonism enhances the tolerability of KarXT, allowing higher doses of xanomeline to be administered for central effects without compromising safety. These mechanistic insights suggest that KarXT offers a unique and promising pharmacological approach for addressing the full spectrum of schizophrenia symptoms.

Muscarinic receptor agonists have shown promising efficacy results in prior trials with single agents. Shekhar *et al.’s* study found that subjects treated with xanomeline performed significantly better than the placebo group in total BPRS and PANSS scores, with notable improvements in the xanomeline group on cognitive test battery measures, particularly in verbal learning and short-term memory function.^24^ Similarly, Krystal *et al.* evaluated emraclidine’s safety for schizophrenia in a two-part study.^25^ The emraclidine groups experienced transient increases in blood pressure and heart rate, which diminished by week 6 and were deemed clinically insignificant. The authors concluded that emraclidine warrants further investigation as a once-daily treatment with a favorable side-effect profile, requiring no titration.

The safety profile of KarXT indicated a distinct pattern of adverse effects consistent with muscarinic receptor agonism, particularly gastrointestinal disturbances. The increased risk of constipation and nausea aligns with known anticholinergic effects, suggesting a mechanistic link to muscarinic activation. Notably, while constipation demonstrated consistent risk across studies, vomiting and dyspepsia exhibited substantial variability, possibly reflecting differences in dosing or patient tolerance. The lack of heterogeneity in SAE and headaches reinforces the absence of systemic or life-threatening risks associated with KarXT, a critical consideration for its tolerability in schizophrenia treatment. However, the moderate-to-high heterogeneity observed in outcomes like vomiting and dyspepsia warrants cautious interpretation, as it may signal context-dependent tolerability influenced by patient characteristics or adjunct therapies. Clinically, these findings highlight the need for proactive monitoring of gastrointestinal symptoms in patients receiving KarXT, particularly during dose titration. The absence of elevated SAE supports its relative safety, though the trade-off between efficacy and tolerability of muscarinic agents must be contextualized within individual patient risk profiles. Further research is needed to elucidate factors contributing to inter-study variability in adverse effects, such as pharmacogenomic influences or long-term safety outcomes.

The findings of this review position KarXT as a potentially transformative treatment for schizophrenia, particularly in addressing negative and cognitive symptoms. Previous study revealed while current antipsychotics are effective in managing positive symptoms, their side effect profiles are concern to some extent, including EPS, metabolic disturbances, and sedation. Additionally, they offer limited efficacy for negative and cognitive symptoms, which are major contributors to disability and poor functional outcomes.^26^ A post hoc analyses has showed that KarXT significantly improves cognitive performance in schizophrenia, particularly in patients with baseline cognitive impairments (LS mean difference = 0.50, *P* = .03; Cohen’s d = 0.50), independent of psychotic symptom reduction. KarXT’s ability to improve a broad range of symptoms without inducing EPS or significant metabolic effects is a significant advancement. Its cholinergic mechanism of action offers an alternative pathway for managing schizophrenia, particularly for patients who do not respond to or cannot tolerate traditional antipsychotics. However, direct head-to-head comparisons with existing therapies are needed to fully contextualize its efficacy and safety relative to established treatment options.

While this review provides valuable insights, several limitations must be acknowledged. First, the number of included studies was small, and all were conducted in the United States, which may limit the generalizability of the findings to other populations. Second, the moderate heterogeneity observed in some efficacy outcomes, such as the Marder negative factor score, indicates the need for additional studies to confirm these findings in diverse patient populations and clinical settings. The safety analysis, while comprehensive, relied on data from a limited number of trials, and long-term safety outcomes remain uncertain. Future research should include larger, multi-center trials with extended follow-up periods to better characterize the long-term efficacy and safety profile of KarXT. Additionally, exploring the impact of KarXT on cognitive symptoms, functional outcomes, and quality of life could provide a more comprehensive understanding of its therapeutic potential. All studies short term (5 weeks) and were conducted in acutely exacerbated patients, limiting the ability to distinguish between primary and secondary negative symptoms. Moreover, all three trials included in this meta-analysis were funded by Karuna Therapeutics, the developer of KarXT. While RCTs represent the gold standard for evaluating efficacy, the reliance on sponsor-funded studies introduces potential biases that warrant consideration.^27^

## CONCLUSION

KarXT (xanomeline–trospium) shows significant promise in treating schizophrenia, improving positive, negative, and cognitive symptoms with a manageable safety profile. Its novel mechanism offers a unique alternative to traditional antipsychotics. Further research is needed to confirm its long-term benefits and broad applicability.

## Supplementary Material

SUPPLEMENTARY_MATERIALS_2_pyaf045

## Data Availability

The data is with the authors and available on request.
