# Supplementary material for: Efficacy and safety of the muscarinic receptor agonist KarXT (Xanomeline-Trospium) in schizophrenia: a systematic review, meta-analysis and Bayesian sensitivity analysis
Source: Int J Neuropsychopharmacol. 2025 Jul 7;29(6):pyaf045. doi: 10.1093/ijnp/pyaf045 (PMC13278781; doi:10.1093/ijnp/pyaf045)
Supplement: SUPPLEMENTARY_MATERIALS_2_pyaf045 [file supplementary_materials_2_pyaf045.docx]

**SUPPLEMENTARY MATERIALS**

**Table S1.** PRISMA Checklist

| **Section and Topic** | **Item #** | **Checklist item** | **Location where item is reported** |
| --- | --- | --- | --- |
| **TITLE** | | |  |
| Title | 1 | Identify the report as a systematic review. | 1 |
| **ABSTRACT** | | |  |
| Abstract | 2 | See the PRISMA 2020 for Abstracts checklist. (made as per the Journal guidelines) | 2 |
| **INTRODUCTION** | | |  |
| Rationale | 3 | Describe the rationale for the review in the context of existing knowledge. | 3 |
| Objectives | 4 | Provide an explicit statement of the objective(s) or question(s) the review addresses. | 3 |
| **METHODS** | | |  |
| Eligibility criteria | 5 | Specify the inclusion and exclusion criteria for the review and how studies were grouped for the syntheses. | 3 |
| Information sources | 6 | Specify all databases, registers, websites, organisations, reference lists and other sources searched or consulted to identify studies. Specify the date when each source was last searched or consulted. | 3 |
| Search strategy | 7 | Present the full search strategies for all databases, registers and websites, including any filters and limits used. | Table S2 |
| Selection process | 8 | Specify the methods used to decide whether a study met the inclusion criteria of the review, including how many reviewers screened each record and each report retrieved, whether they worked independently, and if applicable, details of automation tools used in the process. | 4 |
| Data collection process | 9 | Specify the methods used to collect data from reports, including how many reviewers collected data from each report, whether they worked independently, any processes for obtaining or confirming data from study investigators, and if applicable, details of automation tools used in the process. | 4 |
| Data items | 10a | List and define all outcomes for which data were sought. Specify whether all results that were compatible with each outcome domain in each study were sought (e.g., for all measures, time points, analyses), and if not, the methods used to decide which results to collect. | 4, Table 1 |
|  | 10b | List and define all other variables for which data were sought (e.g., participant and intervention characteristics, funding sources). Describe any assumptions made about any missing or unclear information. | 4 |
| Study risk of bias assessment | 11 | Specify the methods used to assess risk of bias in the included studies, including details of the tool(s) used, how many reviewers assessed each study and whether they worked independently, and if applicable, details of automation tools used in the process. | 4, |
| Effect measures | 12 | Specify for each outcome the effect measure(s) (e.g. risk ratio, mean difference) used in the synthesis or presentation of results. | 5 |
| Synthesis methods | 13a | Describe the processes used to decide which studies were eligible for each synthesis (e.g. tabulating the study intervention characteristics and comparing against the planned groups for each synthesis (item #5)). | 5, Table 1 |
|  | 13b | Describe any methods required to prepare the data for presentation or synthesis, such as handling of missing summary statistics, or data conversions. | NA |
|  | 13c | Describe any methods used to tabulate or visually display results of individual studies and syntheses. | 5 |
|  | 13d | Describe any methods used to synthesize results and provide a rationale for the choice(s). If meta-analysis was performed, describe the model(s), method(s) to identify the presence and extent of statistical heterogeneity, and software package(s) used. | 5 |
|  | 13e | Describe any methods used to explore possible causes of heterogeneity among study results (e.g. subgroup analysis, meta-regression). | 5 |
|  | 13f | Describe any sensitivity analyses conducted to assess robustness of the synthesized results. | 5 |
| Reporting bias assessment | 14 | Describe any methods used to assess risk of bias due to missing results in a synthesis (arising from reporting biases). | NA |
| Certainty assessment | 15 | Describe any methods used to assess certainty (or confidence) in the body of evidence for an outcome. | NA |
| **RESULTS** | | |  |
| Study selection | 16a | Describe the results of the search and selection process, from the number of records identified in the search to the number of studies included in the review, ideally using a flow diagram. | 5, Figure-1, 2 |
|  | 16b | Cite studies that might appear to meet the inclusion criteria, but which were excluded, and explain why they were excluded. | 5, Table 1 |
| Study characteristics | 17 | Cite each included study and present its characteristics. | Table-1 |
| Risk of bias in studies | 18 | Present assessments of risk of bias for each included study. | Figure 2 |
| Results of individual studies | 19 | For all outcomes, present, for each study: (a) summary statistics for each group (where appropriate) and (b) an effect estimate and its precision (e.g. confidence/credible interval), ideally using structured tables or plots. | Table 1, 3 |
| Results of syntheses | 20a | For each synthesis, briefly summarise the characteristics and risk of bias among contributing studies. | 5 |
|  | 20b | Present results of all statistical syntheses conducted. If meta-analysis was done, present for each the summary estimate and its precision (e.g. confidence/credible interval) and measures of statistical heterogeneity. If comparing groups, describe the direction of the effect. | 5, 6 |
|  | 20c | Present results of all investigations of possible causes of heterogeneity among study results. | 5, 6 |
|  | 20d | Present results of all sensitivity analyses conducted to assess the robustness of the synthesized results. | 7 |
| Reporting biases | 21 | Present assessments of risk of bias due to missing results (arising from reporting biases) for each synthesis assessed. | NA |
| Certainty of evidence | 22 | Present assessments of certainty (or confidence) in the body of evidence for each outcome assessed. | Table S3 |
| **DISCUSSION** | | |  |
| Discussion | 23a | Provide a general interpretation of the results in the context of other evidence. | 8 |
|  | 23b | Discuss any limitations of the evidence included in the review. | 8 |
|  | 23c | Discuss any limitations of the review processes used. | 8 |
|  | 23d | Discuss implications of the results for practice, policy, and future research. | 9 |
| **OTHER INFORMATION** | | |  |
| Registration and protocol | 24a | Provide registration information for the review, including register name and registration number, or state that the review was not registered. | 4 |
|  | 24b | Indicate where the review protocol can be accessed, or state that a protocol was not prepared. | 4 |
|  | 24c | Describe and explain any amendments to information provided at registration or in the protocol. | NA |
| Support | 25 | Describe sources of financial or non-financial support for the review, and the role of the funders or sponsors in the review. | 10 |
| Competing interests | 26 | Declare any competing interests of review authors. | 10 |
| Availability of data, code and other materials | 27 | Report which of the following are publicly available and where they can be found: template data collection forms; data extracted from included studies; data used for all analyses; analytic code; any other materials used in the review. | 10 |

**Table S2.** The adjusted search terms as per searched electronic databases

| Database | Search Query | Results |
| --- | --- | --- |
|  | | |
| PubMed | ((("xanomeline" [Supplementary Concept]) OR "trospium chloride" [Supplementary Concept]) OR ("xanomeline–trospium")) AND (("Schizophrenic Disorder") OR (Schizophrenia)) | 50 |
|  | | |
| Embase | ('xanomeline':ab,ti OR 'trospium chloride':ab,ti OR 'xanomeline-trospium':ab,ti) AND ('schizophrenic disorder':ab,ti OR 'schizophrenia':ab,ti) OR (('xanomeline' OR 'trospium chloride' OR 'xanomeline-trospium') AND ('schizophrenic disorder' OR 'schizophrenia'/exp)) | 268 |
|  | | |
| Web of Science | (("xanomeline" OR "trospium chloride" OR "xanomeline–trospium") AND ("Schizophrenic Disorder" OR "Schizophrenia")) | 185 |

**
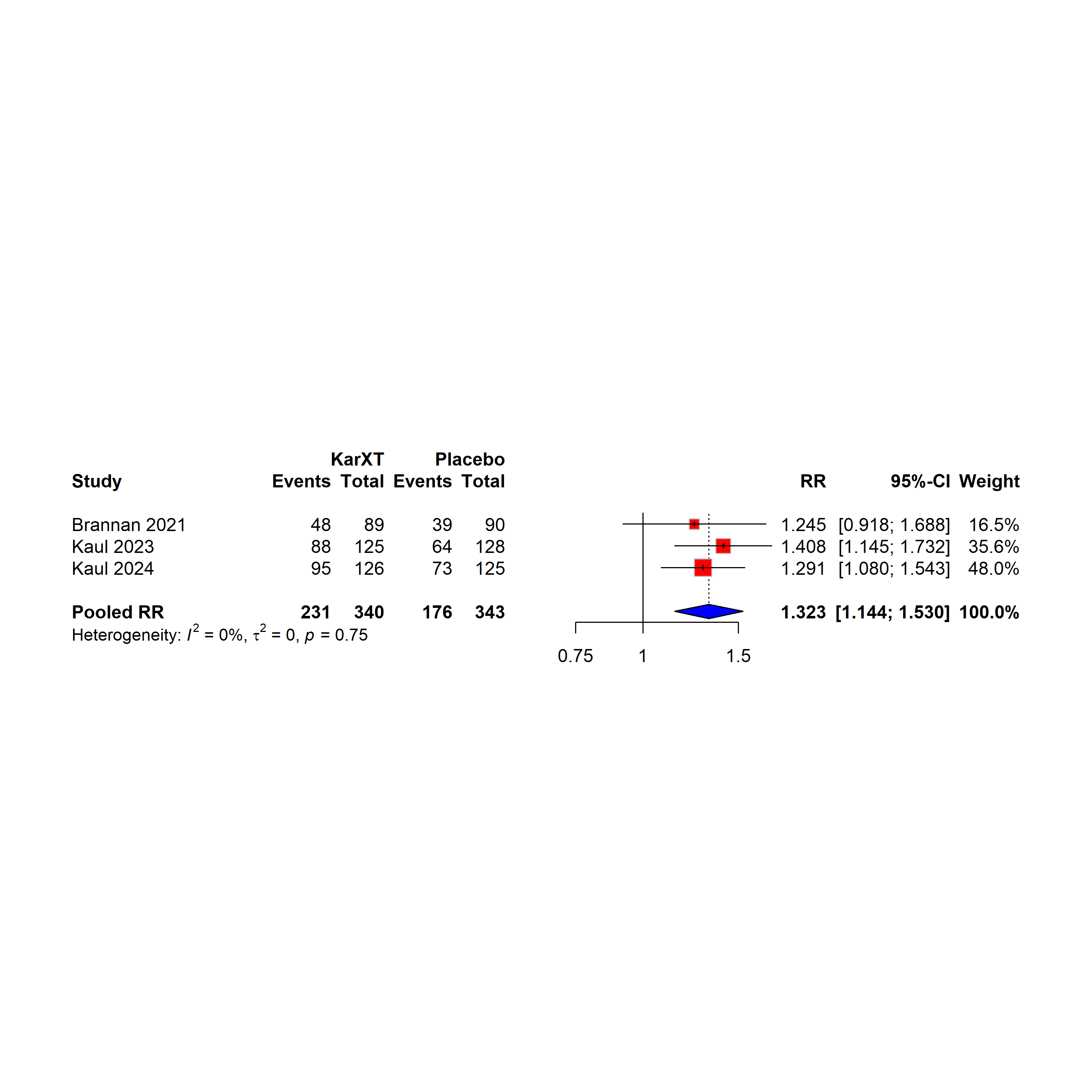
**

**Figure S3: Sensitivity analysis**

**Table S3**: Excluded studies during the full text screening phase

| **Author and year** | **Title** | **Reason for exclusion** |
| --- | --- | --- |
| DeLisi 2024 (1) | A lifetime of mental health treatments for people with schizophrenia: update and narrative review | Reviews |
| Komatsu 2024 (2) | Advancements in Non-Dopaminergic Treatments for Schizophrenia: A Systematic Review of Pipeline Developments | Reviews |
| Luca 2024 (3) | A Clinically Oriented Review of New Antipsychotics for Schizophrenia | Reviews |
| Palma 2024 (4) | Muscarinic control of cardiovascular function in humans: a review of current clinical evidence | Reviews |
| Paul 2024 (5) | Muscarinic Receptor Activators as Novel Treatments for Schizophrenia | Reviews |
| Sutera 2024 (6) | Xanomeline-Trospium in schizophrenia: A detailed review and comparison with the Institute for Clinical and Economic Review's analysis | Reviews |
| Syed (7) | Xanomeline/Trospium Chloride: First Approval | Reviews |
| Tsapakis 2023 (8) | Novel Compounds in the Treatment of Schizophrenia-A Selective Review | Reviews |
| Singh 2022 (9) | Expanding the Neural Network and Utilizing Multi-Targeted Pharmacotherapy to Obtain Optimal Recovery for Complex Multi-Factorial Heterogenous Schizophrenia | Editorial/ or Comments |
| Pomara 2009 (10) | Reduction in muscarinic M1-mediated hypercholinergic state and beneficial cognitive effects of muscarinic agonists in schizophrenia | Editorial/ or Comments |
| Hasan 2024 (11) | Cobenfy (Xanomeline-Trospium Chloride): A New Frontier in Schizophrenia Management | Editorial/ or Comments |
| Shekhar 2008 (12) | Selective muscarinic receptor agonist xanomeline as a novel treatment approach for schizophrenia | Case-reports |
| Tunc 2019 (13) | An unusual case of urge incontinence due to aripiprazole | Case-reports |
| Van Amelsvoort 2018 (14) | Muscarinic M1 receptor signalling underlying cognition in psychotic disorders | Case-reports |
| Hung (15) | Differential effects of Xanomeline-trospium chloride on positive and negative symptoms in Schizophrenia: The role of dose | Case-reports |
| Fabiano (16) | On the potential of xanomeline-trospium chloride for schizophrenia and beyond | Case-reports |
| Andersen 2015 (17) | Antipsychotic-like effect of the muscarinic acetylcholine receptor agonist BuTAC in non-human primates | Outcome not of interest |
| Bradford 2010 (18) | Validation and pharmacological characterisation of MK-801-induced locomotor hyperactivity in BALB/C mice as an assay for detection of novel antipsychotics | Outcome not of interest |
| Cieslik 2017 (19) | The involvement of GABAB receptors in antipsychotic-like effects of positive allosteric modulator of muscarinic acetylcholine M4 receptors. | Outcome not of interest |
| Dencker 2011 (20) | Involvement of a subpopulation of neuronal M4 muscarinic acetylcholine receptors in the antipsychotic-like effects of the M1/M4 preferring muscarinic receptor agonist xanomeline | Outcome not of interest |
| Hellman 2020 (21) | Discovery of Procognitive Antipsychotics by Combining Muscarinic M1 Receptor Structure-Activity Relationship with Systems Response Profiles in Zebrafish Larvae | Outcome not of interest |
| Jones 2012 (22) | Muscarinic and nicotinic acetylcholine receptor agonists and allosteric modulators for the treatment of schizophrenia | Outcome not of interest |
| [Alan Breier](https://pubmed.ncbi.nlm.nih.gov/?sort=date&term=Breier+A&cauthor_id=37036495) 2023 (23) | Evidence of trospium's ability to mitigate cholinergic adverse events related to xanomeline: phase 1 study results | Outcome not of interest |
| [Colin Sauder](https://pubmed.ncbi.nlm.nih.gov/?sort=date&term=Sauder+C&cauthor_id=36414626) 2022 (24) | Effectiveness of KarXT (xanomeline-trospium) for cognitive impairment in schizophrenia: post hoc analyses from a randomised, double-blind, placebo-controlled phase 2 study | Outcome not of interest |
| [Anantha](https://pubmed.ncbi.nlm.nih.gov/?sort=date&term=Shekhar+A&cauthor_id=18593778) 2008 (25) | Selective muscarinic receptor agonist xanomeline as a novel treatment approach for schizophrenia | Outcome not of interest |
| Woolley 2009 (26) | Attenuation of amphetamine-induced activity by the non-selective muscarinic receptor agonist, xanomeline, is absent in muscarinic M4 receptor knockout mice and attenuated in muscarinic M1 receptor knockout mice | Outcome not of interest |
| Sumiyoshi 2013 (27) | Discovery of novel N-substituted oxindoles as selective M1 and M4 muscarinic acetylcholine receptors partial agonists. | Outcome not of interest |
| Sur 2003 (28) | N-desmethylclozapine, an allosteric agonist at muscarinic 1 receptor, potentiates N-methyl-D-aspartate receptor activity | Outcome not of interest |
| Thorn 2017 (29) | Effects of M1 and M4 activation on excitatory synaptic transmission in CA1 | Outcome not of interest |
| Vasiliu 2023 (30) | Next-generation antipsychotics- Trends and perspectives beyond dopaminergic and glutamatergic agents | Outcome not of interest |
| Sellin 2008 (31) | Muscarinic agonists for the treatment of cognition in schizophrenia | Case-series |
| Dean (32) | Muscarinic M1 and M4 receptor agonists for schizophrenia: promising candidates for the therapeutic arsenal | Case-series |

1. DeLisi LE. A lifetime of mental health treatments for people with schizophrenia: update and narrative review. Curr Opin Psychiatry. 2024;37(3):140-6.

2. Komatsu Y, Takehara M, Hart X, Takahashi Y, Hori S, Ueno F, Uchida H. Advancements in Non-Dopaminergic Treatments for Schizophrenia: A Systematic Review of Pipeline Developments. Pharmacopsychiatry. 2024;57(5):221-31.

3. Luca M, Luca A, Serretti A. A Clinically Oriented Review of New Antipsychotics for Schizophrenia. Neuropsychiatr Dis Treat. 2024;20:2637-49.

4. Palma JA. Muscarinic control of cardiovascular function in humans: a review of current clinical evidence. Clin Auton Res. 2024;34(1):31-44.

5. Paul SM, Yohn SE, Brannan SK, Neugebauer NM, Breier A. Muscarinic Receptor Activators as Novel Treatments for Schizophrenia. Biol Psychiatry. 2024;96(8):627-37.

6. Sutera N. Xanomeline-Trospium in schizophrenia: A detailed review and comparison with the Institute for Clinical and Economic Review's analysis. J Manag Care Spec Pharm. 2024;30(6):629-32.

7. Syed YY. Xanomeline/Trospium Chloride: First Approval. Drugs. 2025;85(1):103-9.

8. Tsapakis EM, Diakaki K, Miliaras A, Fountoulakis KN. Novel Compounds in the Treatment of Schizophrenia-A Selective Review. Brain Sci. 2023;13(8).

9. Singh AN. Expanding the Neural Network and Utilizing Multi-Targeted Pharmacotherapy to Obtain Optimal Recovery for Complex Multi-Factorial Heterogenous Schizophrenia. International Medical Journal. 2022;29(3):166-9.

10. Pomara N. Reduction in muscarinic M1-mediated hypercholinergic state and beneficial cognitive effects of muscarinic agonists in schizophrenia. Am J Psychiatry. 2009;166(1):111; author reply -3.

11. Hasan AH, Abid MA. Cobenfy (Xanomeline-Trospium Chloride): A New Frontier in Schizophrenia Management. Cureus. 2024;16(10):e71131.

12. Shekhar A, Potter WZ, Lightfoot J, Lienemann J, Dubé S, Mallinckrodt C, et al. Selective muscarinic receptor agonist xanomeline as a novel treatment approach for schizophrenia. American Journal of Psychiatry. 2008;165(8):1033-9.

13. Tunc S, Buyuksandalyaci E, Basbug HS. An unusual case of urge incontinence due to aripiprazole. European Psychiatry. 2019;56:S480.

14. Van Amelsvoort T, Bakker G, Vingerhoets C, Sahakian BJ, Bloemen O, Caan M, Booij J. Muscarinic M1 receptor signalling underlying cognition in psychotic disorders. Schizophrenia Bulletin. 2018;44:S275-S6.

15. Hung TY, Hsu YC, Chen YCB, Hsu CW. Differential effects of Xanomeline-trospium chloride on positive and negative symptoms in Schizophrenia: The role of dose. EUROPEAN NEUROPSYCHOPHARMACOLOGY. 2025;93:22-3.

16. Fabiano N, Wong S, Zhou C, Correll CU, Hojlund M, Solmi M. On the potential of xanomeline-trospium chloride for schizophrenia and beyond. EUROPEAN NEUROPSYCHOPHARMACOLOGY. 2025;94:17-8.

17. Andersen MB, Croy CH, Dencker D, Werge T, Bymaster FP, Felder CC, Fink-Jensen A. Antipsychotic-like effect of the muscarinic acetylcholine receptor agonist BuTAC in non-human primates. PLoS ONE. 2015;10(4).

18. Bradford AM, Savage KM, Jones DNC, Kalinichev M. Validation and pharmacological characterisation of MK-801-induced locomotor hyperactivity in BALB/C mice as an assay for detection of novel antipsychotics. Psychopharmacology. 2010;212(2):155-70.

19. Cieslik P, Wozniak M, Pilc A, Wierohska J. The involvement of GABAB receptors in antipsychotic-like effects of positive allosteric modulator of muscarinic acetylcholine M4 receptors. European Neuropsychopharmacology. 2017;27:S965-S6.

20. Dencker D, Wörtwein G, Weikop P, Jeon J, Thomsen M, Sager TN, et al. Involvement of a subpopulation of neuronal M4 muscarinic acetylcholine receptors in the antipsychotic-like effects of the M1/M4 preferring muscarinic receptor agonist xanomeline. Journal of Neuroscience. 2011;31(16):5905-8.

21. Hellman K, Ohlsson J, Malo M, Olsson R, Ek F. Discovery of Procognitive Antipsychotics by Combining Muscarinic M1 Receptor Structure-Activity Relationship with Systems Response Profiles in Zebrafish Larvae. ACS Chemical Neuroscience. 2020;11(2):173-83.

22. Jones CK, Byun N, Bubser M. Muscarinic and nicotinic acetylcholine receptor agonists and allosteric modulators for the treatment of schizophrenia. Neuropsychopharmacology. 2012;37(1):16-42.

23. Breier A, Brannan SK, Paul SM, Miller AC. Evidence of trospium's ability to mitigate cholinergic adverse events related to xanomeline: phase 1 study results. Psychopharmacology (Berl). 2023;240(5):1191-8.

24. Sauder C, Allen LA, Baker E, Miller AC, Paul SM, Brannan SK. Effectiveness of KarXT (xanomeline-trospium) for cognitive impairment in schizophrenia: post hoc analyses from a randomised, double-blind, placebo-controlled phase 2 study. Transl Psychiatry. 2022;12(1):491.

25. Shekhar A, Potter WZ, Lightfoot J, Lienemann J, Dubé S, Mallinckrodt C, et al. Selective muscarinic receptor agonist xanomeline as a novel treatment approach for schizophrenia. Am J Psychiatry. 2008;165(8):1033-9.

26. Woolley ML, Carter HJ, Gartlon JE, Watson JM, Dawson LA. Attenuation of amphetamine-induced activity by the non-selective muscarinic receptor agonist, xanomeline, is absent in muscarinic M4 receptor knockout mice and attenuated in muscarinic M1 receptor knockout mice. European Journal of Pharmacology. 2009;603(1-3):147-9.

27. Sumiyoshi T, Enomoto T, Takai K, Takahashi Y, Konishi Y, Uruno Y, et al. Discovery of novel N-substituted oxindoles as selective M1 and M4 muscarinic acetylcholine receptors partial agonists. ACS Medicinal Chemistry Letters. 2013;4(2):244-8.

28. Sur C, Mallorga PJ, Wittmann M, Jacobson MA, Pascarella D, Williams JB, et al. N-desmethylclozapine, an allosteric agonist at muscarinic 1 receptor, potentiates N-methyl-D-aspartate receptor activity. Proceedings of the National Academy of Sciences of the United States of America. 2003;100(23):13674-9.

29. Thorn CA, Popiolek M, Stark E, Edgerton JR. Effects of M1 and M4 activation on excitatory synaptic transmission in CA1. Hippocampus. 2017;27(7):794-810.

30. Vasiliu O, Mangalagiu AG, Petrescu BM, Candea CA, Tudor C, Ungureanu D, et al. Next-generation antipsychotics- Trends and perspectives beyond dopaminergic and glutamatergic agents. European Psychiatry. 2023;66:S558-S9.

31. Sellin AK, Shad M, Tamminga C. Muscarinic agonists for the treatment of cognition in schizophrenia. CNS Spectrums. 2008;13(11):985-96.

32. Dean B. Muscarinic M1 and M4 receptor agonists for schizophrenia: promising candidates for the therapeutic arsenal. EXPERT OPINION ON INVESTIGATIONAL DRUGS. 2023;32(12):1113-21.
